# Preoperative anaemia in distal pancreatectomy: a propensity-score matched analysis

**DOI:** 10.1007/s00423-024-03300-4

**Published:** 2024-04-11

**Authors:** Olga Radulova-Mauersberger, Felix von Bechtolsheim, Christian Teske, Sebastian Hempel, Louisa Kroesen, Mathieu Pecqueux, Christoph Kahlert, Jürgen Weitz, Marius Distler, Florian Oehme

**Affiliations:** 1 Department of Visceral, Thoracic and Vascular Surgery, Faculty of Medicine and University Hospital Carl Gustav Carus, Technische Universität Dresden, Fetscherstrasse 74, 01307 Dresden, Germany; 2 National Center for Tumor Diseases (NCT/UCC), Dresden, Germany

**Keywords:** Pancreatic disease, Anaemia, Distal pancreatic resection, Blood transfusion, CCI

## Abstract

**Background:**

Preoperative anaemia is a prevalent morbidity predictor that adversely affects short- and long-term outcomes of patients undergoing surgery. This analysis aimed to investigate preoperative anaemia and its detrimental effects on patients after distal pancreatectomy.

**Material and methods:**

The present study was a propensity-score match analysis of 286 consecutive patients undergoing distal pancreatectomy. Patients were screened for preoperative anaemia and classified according to WHO recommendations. The primary outcome measure was overall morbidity. The secondary endpoints were in-hospital mortality and rehospitalization.

**Results:**

The preoperative anaemia rate before matching was 34.3% (98 patients), and after matching a total of 127 patients (non-anaemic 42 vs. anaemic 85) were included. Anaemic patients had significantly more postoperative major complications (54.1% vs. 23.8%; *p* < 0.01), a higher comprehensive complication index (26.2 vs. 4.3; *p* < 0.01), and higher in-hospital mortality rate (14.1% vs. 2.4%; *p* = 0.04).

Multivariate regression analysis confirmed these findings and identified preoperative anaemia as a strong independent risk factor for postoperative major morbidity (OR 4.047; 95% CI: 1.587–10.320; *p* < 0.01).

**Conclusion:**

The current propensity-score matched analysis strongly considered preoperative anaemia as a risk factor for major complications following distal pancreatectomy. Therefore, an intense preoperative anaemia workup should be increasingly prioritised.

## Introduction

Pancreatic resection is a complex procedure usually performed in a highly vulnerable cohort of patients. The combination of preoperative morbidity, aggressiveness of the underlying disease, and a high postoperative complication rate is more pronounced in pancreatic surgery than in any other surgical field.

Of note, distal pancreatectomy, one of the two main surgical procedures in pancreatic surgery, has traditionally been considered less complicated, with a lower in-house mortality rate. However, major complications in patients with distal pancreatectomy are high and correlated with poor oncological outcome due to a delay or omission of adjuvant treatment [1, 2]. There is a clear association between postoperative complications and poor OS, as postoperative complications prevent the implementation of mandatory adjuvant therapies [3]. Thus, assessing and treating key preoperative morbidity predictors are crucial in pancreatic surgery, particularly in distal pancreatectomy [3].

The foremost among these preoperative morbidity predictors is the patient’s preoperative haemoglobin (HB) value and the magnitude of preoperative anaemia. Preoperative anaemia is a very prevalent predictor of postoperative morbidity and is present in 25–50% of all surgical patients [4–6], with iron deficiency anaemia (IDA) being the most common cause (25–30%) [7]. The detrimental effects of untreated preoperative anaemia have been well studied and include a significantly longer hospital stay [8], higher in-hospital mortality rates [9], 12–14-fold increased 30-day mortality rates [4], and a significantly increased perioperative transfusion probability [10]. The increased perioperative transfusion probability is associated with a more pronounced negative impact on long-term survival in patients receiving pancreaticoduodenectomy (PD) [11]. This effect could be observed in patients with benign or malignant underlying diseases [12].

Despite the proven negative effects of preoperative anaemia, its diagnosis and treatment are undervalued in routine clinical practice [8, 13]. Preoperative anaemia is still seen merely as an indicator of the severity of the underlying disease, and the treatment options are not yet sufficiently known to all treatment providers [13]. In particular, considering the ease of preoperative diagnosis and treatment [7] of IDA, it is surprising that current standards are only present at individual hospitals [14].

Therefore, the influence of preoperative anaemia on the course of our patients after pancreatectomy should be the focus of future research. Only one trial [11] has discussed preoperative anaemia as a morbidity predictor in distal pancreatectomy. Consequently, there is still a lack of evidence regarding whether anaemia influences the postoperative complication rate and long-term prognosis of patients undergoing distal pancreatectomy.

For this reason, we investigated how the presence of preoperative anaemia altered the outcome for patients with benign or malignant pancreatic disease in a large cohort from a single institution in a propensity-score matching analysis. This may help to identify and optimise clinical targets during the perioperative care period to improve the overall prognosis of patients scheduled for pancreatic surgery.

## Methods

This article was written following the Strengthening for the Reporting of Observational Studies in Epidemiology Statement (STROBE) [15]. The experimental protocol of the study was approved by the local ethics committee of the TU Dresden (decision number EK 576122019). All experimental methods were carried out in accordance with relevant guidelines.

### Patients

This study was a propensity-score matching analysis of all consecutive patients (18 years or older) who underwent distal pancreatectomy for pancreatic malignancies, such as ductal adenocarcinoma (PDAC), neuroendocrine malignancy (NET), metastases of renal cell carcinoma, or a cystic pancreatic lesion or a chronic pancreatitis between January 2010 and December 2021 at the Department of Visceral, Thoracic, and Vascular Surgery, University Hospital Carl Gustav Carus, Technische Universität Dresden, Germany. We excluded all patients who underwent emergency resection, enucleation, or palliative procedures.

### Patient data

Patient characteristics included median age, sex, body mass index, and preoperative (insulin)-dependent diabetes mellitus ((N)IDDM). Cardiac risk profiles were assessed for each patient by evaluating their histories of hypertension and myocardial infarction. The Charlson comorbidity index was used to better represent the preoperative morbidity in our cohort [16]. Preoperative morbidity-predicting risk factors, such as smoking, alcohol abuse, or neoadjuvant chemotherapy, were also included.

The indication for surgery was given in terms of existing benign or malignant underlying disease and categorised as malignancy (pancreatic ductal adenocarcinoma [PDAC], neuroendocrine tumour (NET) or benign (e.g., pancreatitis, cystic pancreatic lesion).

Since 2018, we adopted an enhanced recovery program (ERP) in our institution. Postoperatively, patients receive water and tea on the day of operation, and liquid diet (soup, yoghurt) on POD 1. If tolerated, the diet is increased to a normal meal on POD 2 and after DP on POD 1. If drainage is placed intraoperatively, amylase is measured on POD 1 and 3. If the amylase in the drainage is < 40 µmol/l (< 2000 U/ml) and the volume of serous fluid over 24 h is < 800 ml, the drainage is removed on POD 3. The patients are mobilised with physiotherapy care in the evening of the operation day. The patients are discharged on POD 5 or 6 if no complications occur.

### Anaemia, blood transfusion rate, and postoperative HB course

Preoperative anaemia was defined for female (< 7.45 mmol/l) and male (< 8.07 mmol/l) patients according to the World Health Organization (WHO) definition [17]. The WHO defines anaemia as a haemoglobin level < 8.07 mmol/l (< 130 g/L) for men and < 7.45 mmol/l (< 120 g/L) for women. No iron substitution or erythropoietin was administered preoperatively.

The perioperative anaemia workup included the percentage of patients with anaemia and those who received blood transfusions during the pre-, intra-, and postoperative courses. The decision for transfusions was based on physician judgement, which recommends adherence to our standard operation procedure (SOP) for transfusions. The SOP follows the recommendations of the “patient blood management movement” and recommends transfusing earliest at an HB value below 4.9 mmol/l (7.9 g/dl) and only in the presence of symptoms [5]. An absolute transfusion trigger was defined as a threshold of 3.7 mmol/l (5.9 g/d) and below where a transfusion is indicated. Another indicator for a transfusion is an acute bleeding, in this case independent of the current HB.

The analyses of postoperative blood parameters included HB values and leukocyte and c-reactive protein (CRP) levels. Postoperative blood sampling was performed on postoperative days (POD) 1, 3, 5, and if patients were still not discharged, on POD 7. During the analysis, the lowest HB value in the postoperative course was defined for the period between POD 1–3, POD 4–7, POD 8–14, and the lowest value during hospitalization.

### Primary outcome: postoperative complication rate

The primary outcome measure was the major complication rate according to grade ≥ 3 of the Clavien–Dindo Classification (CDC) [18]. We incorporated the Comprehensive Complication Index (CCI) to strengthen the statistical analysis [19]. This validated measure based on the CDC is a reliable predictor of the postoperative morbidity and shows a high sensitivity in pancreatic surgery [20].

We additionally analysed post-pancreatectomy specific complications, such as delayed gastric emptying (DGE) [21], postoperative pancreatic fistula (POPF) [22], and post-pancreatectomy haemorrhage (PPH) [23] according to the consensus definitions of the International Study Group of Pancreatic Surgery.

### Secondary outcome: postoperative course, rehospitalization rate, and eligibility for adjuvant chemotherapy

For the postoperative course, total and intensive care unit (ICU) length of stay were considered and defined as the number of days between surgery and discharge from the hospital or the ICU, respectively. The rehospitalization rate was defined as any hospital readmission within 30 days of discharge after the index operation due to complications following distal pancreatectomy. For oncology patients, successful initiation of adjuvant chemotherapy, if indicated, was accounted for.

### Statistical analysis

Data were analysed using SPSS version 26 (IBM Corp., Armonk, NY, USA). The normality of continuous data was tested using the Kolmogorov–Smirnov test, and frequency distributions were examined. The homogeneity of variances was analysed using Levene’s test.

Descriptive statistics were performed to compare baseline characteristics between patients with and without preoperative anaemia and to describe the occurrence of complications and blood administered during the pre-, intra-, and postoperative courses. At the same time, the median and interquartile range (IQR) were used for continuous variables.

Comparative analysis was conducted between patients with and without preoperative anaemia using the chi-square test, Fisher’s exact test, or Mann–Whitney/Student’s-t test, where appropriate.

Logistic regression analysis was performed to determine the correlation between preoperative anaemia and CDC complications ≥ 3. The risk factors were age, sex, comorbidities, preoperative anaemia, pre-, intra-, and postoperative blood transfusions, intraoperative blood loss, lowest postoperative HB value, and decreased HB value during hospitalisation. A multivariate stepwise regression model was used, including all variables with a *p* value < 0.3 in the univariate regression analysis.

A *p* value of < 0.05 was used as the statistical significance threshold for all analyses. The inclusion criterion for analysis was a complete dataset for each patient. Patients with missing data were treated as missing completely at random, and they were excluded from the specific analysis.

The propensity-score matching was performed by SPSS with a matching tolerance of 0.2 in a non-anaemic to anaemic ratio of 1:2. Matching variables were defined by the difference in the baseline characteristics between the cohorts and by the significance of a regression analysis defining predictors of a major postoperative complication before matching in the whole cohort (288 patients with a DP from January 2010 and December 2021). Matching variables had been as follows: sex, neoadjuvant chemotherapy, Charlson comorbidity index, histopathological characteristics, minimal-invasive approach, multivisceral resections/spleen resection, duration of operation, and intraoperative blood loss. The effects of the propensity-score matching analysis are given in Table 1.

**Table 1:** Basic patient characteristics: before and after matching

	Unmatched cohort			Matched cohort		
	*No anaemia*	*Anaemia*	*p Value*	*No anaemia*	*Anaemia*	*p Value*
Patients [*n*]	188	98		42	85	
Sex [*n* (%)]						
M	81 (43.1)	67 (68.4)	** < 0.001**	24 (57.1)	60 (70.6)	0.13
F	107 (56.9)	31 (31.4)		18 (42.9)	25 (29.4)	
Median age [years] (IQR)	64 (56–72)	63 (50–75)	0.79	67 (58.8–78.3)	63 (48.5–75.5)	0.08
Median BMI [kg/m^2^] (IQR)	26 (23.1–29.1)	24.3 (21.8–27.8)	** < 0.01**	26.5 (23.2–30)	24.3 (21.7–27.2)	**0.02**
Charlson comorbidity index [*n*] (IQR)	4 (2–6)	5 (3–7)	0.07	5 (4–7)	5 (3–7)	0.14
Drinking [*n* (%)]	28 (15.1)	21 (22.3)	0.13	10 (23.8)	21 (25.9)	0.8
Smoking [*n* (%)]	39 (21)	28 (29.8)	0.1	11 (26.2)	25 (30.9)	0.59
Hypertension [*n* (%)]	92 (50.8)	51 (56.7)	0.37	24 (57.1)	44 (56.4)	0.94
Diabetes [*n* (%)]	43 (23.2)	26 (27.4)	0.45	16 (38.1)	20 (24.4)	0.11
Insulin-dependent diabetes (IDDM) [n (%)]	19 (10.3)	12 (12.6)	0.55	9 (21.4)	12 (14.6)	0.34
naCTx [*n* (%)]	18 (9.6)	24 (24.5)	** < 0.01**	7 (16.7)	16 (18.8)	0.77
*Histopathological analysis [n (%)]*						
Benign	66 (35.1)	22 (22.4)	** < 0.001**	4 (9.5)	22 (25.9)	0.05
Pancreatic ductal adenocarcinoma (PDAC)	88 (46.8)	38 (38.8)		25 (59.5)	33 (38.8)	
Neuroendocrine tumour (NET)	34 (18.1)	38 (38.8)		13 (31)	30 (35.3)	
*Surgical approach [n (%)]*						
Minimal-invasive	95 (50.5)	18 (18.4)	** < 0.001**	9 (21.4)	16 (18.8)	0.73
Conversion	17 (13.6)	9 (22)	0.2	3 (10.3)	8 (21.6)	0.22
Spleen resected	154 (81.9)	90 (91.8)	**0.02**	35 (83.3)	78 (91.8)	0.15
Multi-visceral resections	21 (11.2)	43 (43.9)	** < 0.001**	9 (21.4)	30 (35.3)	0.11
Duration of operation [min] (IQR)	254 (189–313)	290 (210–365)	** < 0.01**	284 (225–359)	274 (206–353)	0.54
Intraoperative blood loss [ml] (IQR)	400 (138–600)	600 (438–1200)	** < 0.01**	600 (400–1000)	700 (500–1250)	0.21

*IQR* interquartile range, *BMI* body mass index, *naCTx* neoadjuvant chemotherapy, *ASA American Society of Anesthesiologists*

## Results

**Fig. 1:**
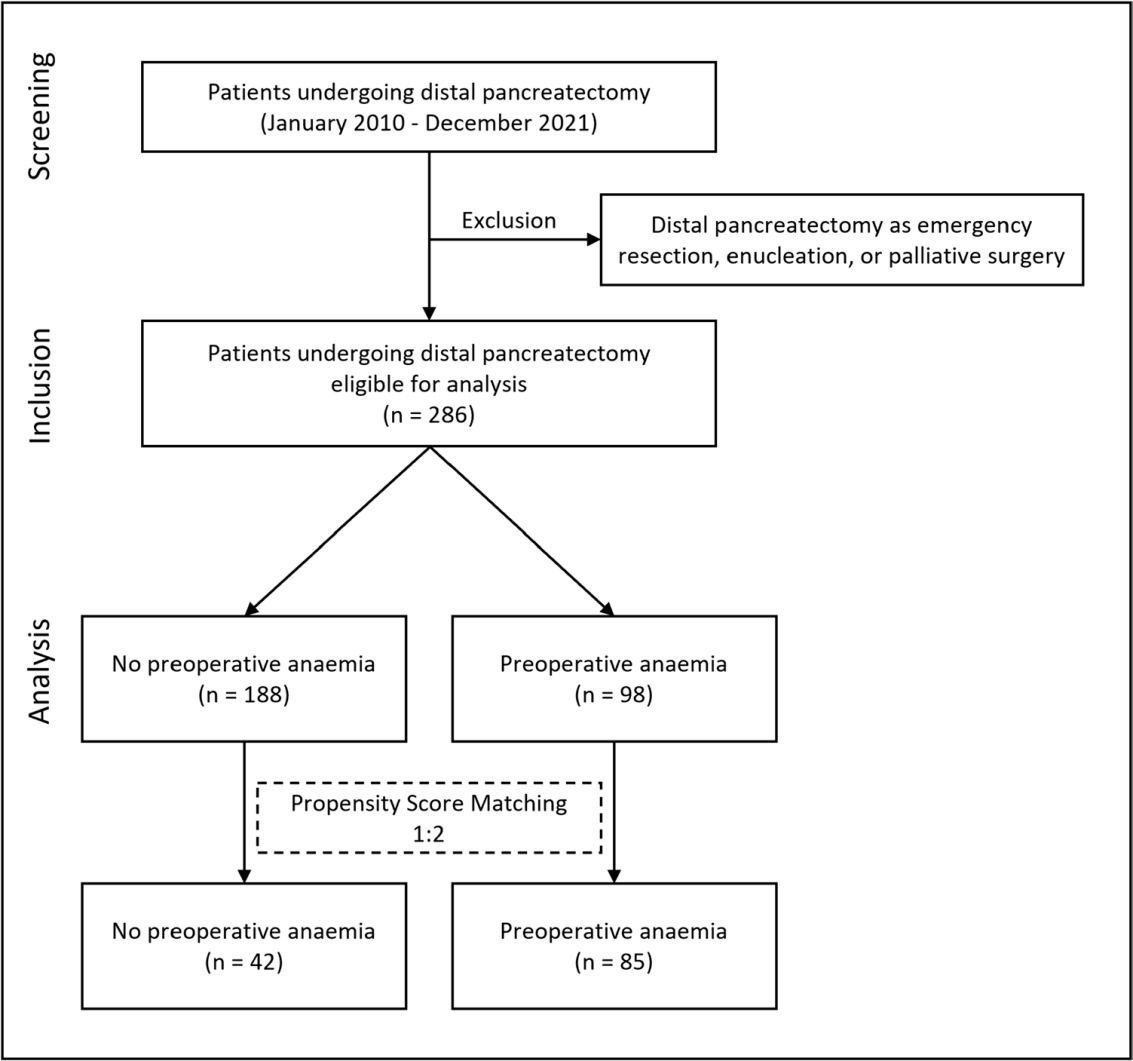
Trial scheme

A total of eligible 286 patients underwent distal pancreatectomy between January 2010 and December 2021 at the University hospital Dresden. After propensity-score matching, a total of 127 patients (42 in the non-anaemic cohort and 85 in the anaemic cohort) remained (Fig. 1).

### Patient cohort

The median age of all patients was 65 (IQR 52–76) years, and the majority (*n* = 58; 45.6%) underwent surgery for PDAC (Table 1). The median Charlson comorbidity index was 5 (IQR 3–7), and 28.3% (*n* = 36) of the patients had preoperative diabetes mellitus. In 25 patients (19.7%), a minimally invasive approach was the preferred type of abdominal access (Table 1).

In total, 85 patients with a preoperative anaemia were selected during the propensity-score matching. The median preoperative HB value was 8.5 mmol/l (13.7 g/dl) (IQR 8.1–9 mmol/l or 13.1–14.5 g/dl) for patients without preoperative anaemia. Patients with preoperative anaemia had an HB value of 6.8 (11 g/dl) mmol/l (IQR 6–7.4 mmol/l or 9.7–11.9 g/dl).

### Effect of the propensity-score matching

Before matching, we found significantly more (*p* < 0.001) male patients with anaemia (67 patients, 68.4%) than female patients. At the same time, patients with preoperative anaemia had a higher rate of neoadjuvant chemotherapy (*p* < 0.01) than patients without preoperative anaemia.

Interestingly, patients with preoperative anaemia were more often operated on using an open approach (*p* < 0.001) and had a higher multi visceral resection rate (*p* < 0.001). The duration of operation and the intraoperative blood loss differed significantly (both *p* < 0.01) between patients with and without anaemia. These differences were eliminated by the propensity-score matching (Table 1).

### Preoperative blood values and postoperative HB course

Preoperative blood samples revealed comparable results between patients with and without anaemia regarding leukocyte count. A statistically significant higher CRP (12.6 mg/L vs. 3.9 mg/l; *p* value < 0.001) and higher INR, (1.01 vs. 1.06; *p* value < 0.01) were found in the cohort of patients with preoperative anaemia.

The postoperative in-hospital HB course showed the expected significant difference between patients with and without preoperative anaemia. The HB value was significantly (*p* < 0.001 for all analysis) lower in the cohort of patients with preoperative anaemia throughout the postoperative course. On the first postoperative day, the HB value of the cohort without preoperative anaemia was 1.1 mmol/l higher than that of patients with preoperative anaemia. This increased to a HB value difference of 1.2 mmol/l from POD 7 (Table 2).

**Table 2:** Perioperative blood results

	*Overall*	*No anaemia*	*Anaemia*	*p Value*
Patients [*n* (%)]	127	42 (33.1)	85 (66.9)	
*Preoperative lab values*				
Haemoglobin [mmol/l] (IQR)	7.4 (6.4–8.1)	8.5 (8.1–9)	6.8 (6–7.4)	** < 0.001**
Haematocrit [%] (IQR)	0.36 (0.32–0.39)	0.41 (0.39–0.43)	0.34 (0.3–0.36)	** < 0.001**
Leucocytes [GPT/l] (IQR)	7.4 (5.9–9.5)	6.8 (5.6–8.6)	7.6 (5.9–10.9)	0.06
Platelets [X1000/mm^3^] (IQR)	244 (184–345)	214.5 (166.3–295.5)	263 (190.5–376)	** < 0.01**
CRP serum [mg/l] (IQR)	6.2 (1.6–36.2)	3.9 (0.6–8.8)	12.6 (2.4–67.4)	** < 0.001**
INR [*n*] (IQR)	1.03 (1–1.1)	1.01 (0.97–1.06)	1.06 (1–1.12)	** < 0.01**
Albumin [g/l] (IQR)	41.9 (38.4–44.6)	44 (40–46.1)	40.8 (34.9–43.8)	** < 0.01**
Preoperative CA 19–9 [U/ml] (IQR)	7.7 (4–67.7)	8.6 (2.9–297.1)	7.5 (4.7–27.9)	0.29
Preoperative CEA [U/ml] (IQR)	2.4 (1.5–4.6)	2.7 (1.6–6.7)	2.4 (1.5–4.2)	0.53
*Postoperative haemoglobin values [mmol/l] (IQR)*				
Day 1	6.4 (5.6–7.1)	7.2 (6.7–7.5)	6.1 (5.3–6.7)	** < 0.001**
Day 3	5.5 (5–6.2)	6.1 (5.5–6.6)	5.4 (5–6)	** < 0.001**
Day 5	6 (5.3–6.5)	6.6 (6.1–7.1)	5.7 (5.1–6.3)	** < 0.001**
Day 7	6.1 (5.2–6.9)	7 (6.1–7.9)	5.8 (5–6.4)	** < 0.001**
Lowest through days 4–7	5.8 (5.1–6.4	6.5 (5.8–7.3)	5.5 (4.8–6.2)	** < 0.001**
Lowest through days 8–14	5.6 (4.6–6.2)	6.4 (5–7.3)	5.2 (4.3–5.9)	** < 0.001**
Lowest through hospital stay	5 (4.4–6)	6 (4.8–6.5)	4.7 (4.2–5.5)	** < 0.001**
*Postoperative CRP values [mg/l] (IQR)*				
Day 1	85.5 (55.2–122.4)	68 (47.2–110.4)	90.9 (60.7–136.8)	**0.04**
Day 3	144.7 (90.1–212.2)	108.4 (61.7–152.9)	157.4 (102–230.9)	** < 0.01**
Highest through days 4–7	121.3 (70.4–171.6)	96.4 (58.3–156.8)	128.1 (78.7–189.6)	0.07
Highest through days 8–14	115.6 (66.2–243.9)	106.3 (42.7–264.9)	118.3 (74.8–234.7)	0.65
*Postoperative leucocytes values [GPT/l] (IQR)*				
Day 1	14.2 (11.9–17.8)	14.6 (12.6–17.7)	13.7 (11.5–18.9)	0.75
Day 3	15.1 (11.6–18.2)	15.5 (11.5–19.1)	15 (11.9–17.9)	0.66
Highest through days 4–7	12.8 (10.9–17.1)	12.1 (10.2–15.3)	13.3 (11.1–18.3)	0.28
Highest through days 8–14	15.6 (11.6–24.2)	15.3 (9.6–21.1)	15.9 (11.9–26.3)	0.12

### Primary outcome: impact of preoperative anaemia on postoperative morbidity

The overall complication rate (for the propensity-score matched cohort) was 67.5% (*n* = 85) with a major complication rate (CDC ≥ 3) of 44.1% (*n* = 56). The median CCI was 20.9 (IQR, 0–26.2), and the in-hospital mortality rate was 10.2% (*n* = 13).

Anaemia was highly correlated with the occurrence of postoperative complications. Patients with preoperative anaemia had a significantly higher major complication rate (54.1% vs. 23.8%; *p* < 0.01) and a significantly higher CCI (26.2 vs. 4.3; *p* < 0.01). Notably, the in-hospital mortality (14.1% vs. 2.4%; *p* = 0.04) and 30-day mortality (11.8% vs. 0%; *p* = 0.03) rates were significantly higher in the cohort with preoperative anaemia (Table 3).

**Table 3:** Outcome analysis

	*Overall*	*No anaemia*	*Anaemia*	*p Value*
Patients [*n* (%)]	127	42 (33.1)	85 (66.9)	
*Postoperative complication*				
Any complication [*n* (%)]	85 (67.5)	21 (50)	64 (76.5)	** < 0.01**
CD ≥ 3 [*n* (%)]	56 (44.1)	10 (23.8)	46 (54.1)	** < 0.01**
In-hospital mortality [*n* (%)]	13 (10.2)	1 (2.4)	12 (14.1)	**0.04**
30-day mortality [*n* (%)]	10 (7.9)	0	10 (11.8)	**0.03**
Comprehensive complication index [*n*] (IQR)	20.9 (0–42.4)	4.3 (0–26.2)	26.2 (0–50.1)	** < 0.01**
Clinical relevant POPF [*n* (%)]	36 (28.3)	8 (19)	28 (32.9)	0.1
PPH [*n* (%)]	11 (8.7)	2 (4.8)	9 (10.6)	0.27
Length of hospital stay (LOS) (IQR)	16 (11–28)	14.5 (10–24.5)	17 (12–30)	0.22
Length of intensive care unit stay (ICU stay) (IQR)	2 (1–5)	1 (0.8–3)	2 (1–6)	0.14
ICU readmission [*n* (%)]	19 (15)	3 (7.1)	16 (18.8)	0.08
Adjuvant CTX received (if indicated) [*n* (%)]	29 (69)	13 (76.5)	16 (64)	0.39
30-Day rehospitalization rate [*n* (%)]	59 (46.5)	21 (50)	38 (44.7)	0.57
*Blood transfusion*				
Intraoperative RBC transfused [*n* (%)]	22 (17.3)	1 (2.4)	21 (24.7)	** < 0.01**
Intraoperative plasma transfused [*n* (%)]	19 (15)	0	19 (22.4)	** < 0.01**
Intraoperative platelets transfused [*n* (%)]	6 (4.7)	0	6 (7.1)	0.18
Postoperative RBC transfused [*n* (%)]	45 (35.4)	4 (9.5)	41 (48.2)	** < 0.001**
Postoperative plasma transfused [*n* (%)]	22 (17.3)	3 (7.1)	19 (22–4)	**0.04**
Postoperative platelets transfused [*n* (%)]	8 (6.3)	1 (2.4)	7 (8.2)	0.27

Regression analysis revealed preoperative anaemia as an independent risk factor. Preoperative anaemia increased the probability of a major postoperative complication by 4.05-fold (95% CI: 1.587–10.320; *p* value < 0.01). The other significant morbidity predictors were prolonged time of surgery and a higher Intraoperative blood loss. However, it did not result in a clinically relevant increase in the risk of postoperative complications (OR 1.001–1.005) (Table 4).

**Table 4:** Prediction of CDC ≥ 3 complication: uni- and multivariate regression

	Univariate analysis			Multivariate analysis		
	Relative OR	95% CI	*p* Value	Relative OR	95% CI	*p* Value
*Patient characteristics*						
Female^2^	1.147	0.546–2.411	0.72	1.084	0.435–2.699	0.86
Age^1^	0.977	0.953–1.001	0.06	0.990	0.956–1.026	0.719
BMI^2^	0.943	0.881–1.010	0.1	0.985	0.908–1.069	0.72
*Preoperative morbidity*						
Drinking^2^	0.543	0.230–1.279	0.16	0.446	0.160–1.245	0.12
Smoking^2^	1.268	0.580–2.768	0.55	n.a		
NIDDM^2^	1.053	0.482–2.299	0.9	n.a		
IDDM^2^	0.763	0.291–1.997	0.58	0.987	0.313–3.115	0.98
Charlson comorbidity score^1^	0.944	0.831–1.071	0.37	0.999	0.822–1.213	0.99
Anaemia^2^	3.774	1.648–8.642	** < 0.01**	4.047	1.587–10.320	** < 0.01**
Malignancy^2^	1.096	0.458–2.619	0.84	n.a		
Neoadjuvant chemotherapy^2^	1.333	0.531–3.348	0.54	0.844	0.273–2.604	0.768
*Perioperative*						
Minimal-invasive procedure^2^	0.812	0.333–1.977	0.65	0.961	0.331–2.794	0.94
Duration of procedure^1^	1.005	1.001–1.008	** < 0.01**	1.005	1.001–1.009	**0.02**
Estimated blood loss^1^	1.001	1–1.002	**0.02**	1.001	1–1.002	**0.04**
Intraoperative RBC^1^	1.664	0.660–4.193	0.28	0.646	0.064–6.476	0.71

^1^Continuous variables

^2^Dichotomic variables

### Impact of preoperative anaemia on perioperative transfusion rate

The overall transfusion rates for intraoperative and postoperative red blood cell (RBC) transfusions were 17.3% and 35.4%, respectively. The frequency of RBC transfusion was significantly higher intraoperatively (24.7% vs. 2.4%; *p* < 0.01) and postoperatively (48.2% vs. 9.5%; *p* < 0.001) in the patient cohort with preoperative anaemia.

### Secondary outcome: postoperative course, rehospitalization rate, and eligibility for adjuvant chemotherapy

Patients with preoperative anaemia remained longer on ICU (1 vs. 2 days; *p* = 0.14) and overall in the hospital (17 vs. 14.5 days; *p* = 0.22). The same cohort also had a higher ICU readmission rate (18.8% vs. 7.1%; *p* = 0.08). The rehospitalization rate was similar in both groups (44.7% vs. 50%; *p* = 0.57) and did not significantly differ between the groups (Table 3). The rate of oncologic patients eligible for adjuvant chemotherapy, if indicated, was comparable between both groups.

## Discussion

Preoperative anaemia is a major, independent, and controllable factor in surgery that poses a significant risk for postoperative complications, irrespective of the underlying disease. This correlation was shown in our group’s first paper concerning anaemia before pancreatic surgery [12] and was confirmed by Pecorelli et al. [24]. Surprisingly, this group could not reproduce these results in patients undergoing distal pancreatectomy but only in those who underwent classic pancreatectomy. Therefore, the primary aim of this study was to strengthen the evidence on the impact of anaemia on the postoperative morbidity profile after distal pancreatectomy.

Our study revealed that anaemia was prevalent in a cohort of patients undergoing distal pancreatectomy. A total of 98 (34.3%) patients were anaemic preoperatively. The only comparable cohort study by Pecorelli et al. [24] described a preoperative anaemia rate of 20.4%. Referring to the published data on anaemia in patients before visceral surgery, international preoperative anaemia rates range from 20 to 50% [5, 25–28]. Therefore, it should be assumed that at least every fifth patient suffers from preoperative anaemia before distal pancreatectomy. Considering that preoperative anaemia is one of the most important and controllable preoperative morbidity predictors [28], it is surprising that the evaluation and therapy of anaemia before surgical interventions are only performed in a minority of patients [29].

Currently, data on the influence of preoperative anaemia on morbidity following distal pancreatectomy are limited. Our study is the first to show the expected detrimental effects of preoperative anaemia in patients undergoing distal pancreatectomy. The only study by Pecorelli et al. did not show any influence of preoperative anaemia on postoperative complications. In contrast, our study showed that patients with preoperative anaemia were significantly more likely to have major postoperative complications. Preoperative anaemia was an isolated risk factor in our analysis and increased the probability of a major postoperative complication by 4.05-fold. These data are consistent with those of international publications on the influence of preoperative anaemia on postoperative morbidity in cardiac surgery [30, 31], orthopaedic surgery [32], and visceral surgery [9, 33, 34].

Our study is the first to report the postoperative course of HB values in patients undergoing distal pancreatectomy. Blood samples were taken at POD 1, 3, 5, and POD 7 in a standardised fashion. These data showed that preoperative anaemia is a simple preoperative morbidity-predicting factor that affects the postoperative course. Patients with preoperative anaemia consistently had a median of 1 mmol/l lower HB value during the postoperative course than patients without preoperative anaemia. The increased likelihood and rate of blood transfusion in the cohort of patients with preoperative anaemia translates into an increased complication rate. In turn, patients with perioperative RBC transfusion are known to have significantly more frequent infectious complications [35, 36]. The main hypothesis for the observed association between blood transfusion and complication rate assumes that RBC transfusion induces transfusion-related immune modulation (TRIM). TRIM leads to immunosuppression, which may explain the impaired recovery of the patient and the increased risk of postoperative complications.

We further found a trend to prolonged ICU stay and LOS in the group with preoperative anaemia. This affects the concept of ERP, we adopted since 2018. As our cohort also includes patients before this period, this could be one possible explanation for these results. However, ERP has been increasingly established in pancreatic surgery to provide fast recovery and cost-effectiveness, and some authors show that it can be affected by postoperative complications [37, 38].

Since preoperative anaemia has an impact on postoperative complications, this also affects the ERP concept, and this should be further addressed in prospective studies.

In our results, we found a significantly increased CRP in patients with anaemia. However, some authors found this condition to be often associated with postoperative inflammatory complications; the relation to anaemia is currently not clear enough [39, 40]. Preoperative anaemia workup must be standard before distal pancreatectomy. Preoperative workup should include ferritin and transferrin level analysis, folic acid and vitamin B12 analysis, and identification of potential coagulopathy [35, 41]. Current data on preoperative iron supplementation in patients with IDA are strong, and iron supplementation should be given to nearly 40% of patients with IDA [6, 7]. The initial consideration of definitive iron supplementation in patients before major abdominal surgery, regardless of IDA status, has been critically changed since the PREVENTT trial [42].

Our data strongly support the urgent clinical need for a standardised introduction of a preoperative anaemia workup in pancreatic surgery to reduce perioperative complication rates and improve clinical outcomes [5].

We acknowledge the limitations of this study, starting with its retrospective design and known and unknown sources of bias. Furthermore, we present a heterogeneous group of patients, which may affect the transferability of the presented results. For example perioperative blood transfusion was unfortunately not categorized by the primary endpoint—postoperative complication—and a logistic regression for perioperative transfusions was not implemented. Further, we did not perform a sensitivity analysis for POPF—one of the main determinant in the onset of major complication, as we focused on the primary endpoint overall morbidity, which may cause some bias. However, this was accounted for in part by using a propensity score matching. On the other hand, our matched data set indicates that the patient cohorts, which were compared, might represent just a selected subset of the original data set of all distal pancreatectomies. As a result, the generalizability of our findings might be compromised and should be interpreted as an outcome potentially based on a certain subset of patients with distal pancreatectomy.

## Conclusion

This study is the first to provide evidence of the significant impact of preoperative anaemia on short- and long-term outcomes after distal pancreatectomy. Preoperative anaemia is very likely to be prevalent in patients undergoing distal pancreatectomy and to be significantly associated with the incidence of major postoperative complications, perioperative transfusions, and worse mortality.

Thus, a preoperative anaemia workup should be strongly considered for patients undergoing distal pancreatectomy. This condition should be moreover understood as an independent but controllable morbidity predictor rather than an inherent circumstance of the underlying disease. We highly recommend implementing and strictly following a defined interdisciplinary preoperative patient blood management protocol.

## Data Availability

The participants of this study did not give written consent for their data to be shared publicly, so due to the sensitive nature of the research supporting data is not available.
